# Announcement: 4^th^ International Conference on Environmental and Biological Aspects of Main Group Organometals (ICEBAMO 98)

**DOI:** 10.1155/MBD.1997.55

**Published:** 1997


					ICEBAMO 98
4th
INTERNATIONAL CONFERENCE ON ENVIRONMENTAL
AND BIOLOGICAL ASPECTS OF MAIN GROUP
ORGANOMETALS
The CEBAMO meetings aim to facilitate interdisciplinary discussion on
environmental and biological aspects of main-group organometals. Previous
meetings in Padua (1986 and 1991) and Bordeaux (1994) have brought together
researchers from the fields of toxicology, environmental sciences, analytical
chemistry, biochemistry, and synthetic organometallic chemistry.
The fourth meeting in the ICEBAMO series will take place in Denmark from 28
June to 2 July 1998 at the Gammel Avernas Conference Centre on the beautiful
island of Funen. The conference will provide a forum for discussion of the
following aspects of organometallic compounds'
Origin and pathways in the environment
Fate in organisms including accumulation and toxicological effects
Biological and biochemical studies on transformation/detoxification processes
Analytical chemistr including analysis of chemical species
Main group metal-based drugs
The meeting will consist of oral presentations and posters with a strong emphasis
on scientific discussion. Accordingly, ample time for questions and discussion
will be made available both during the formal sessions and afterwards while
relaxing in the picturesque surroundings of Gammel Averns.
A detailed announcement and call for abstracts will be released in July 1997.
Meanwhile, further information may be obtained from:
Kevin A. Francesconi
Institute of Biology
Odense University
DK-5230 Odense M
Denmark
Fax" (45) 65 93 04 57
E-mail" kaf@biology.ou.dk

				

